# Functional Identification and Characterization of the *Brassica Napu*s Transcription Factor Gene *BnAP2*, the Ortholog of *Arabidopsis Thaliana APETALA2*

**DOI:** 10.1371/journal.pone.0033890

**Published:** 2012-03-27

**Authors:** Xiaohong Yan, Lei Zhang, Bo Chen, Zhiyong Xiong, Chunli Chen, Lijun Wang, Jingyin Yu, Changming Lu, Wenhui Wei

**Affiliations:** 1 Key Laboratory of Oil Crop Biology and Genetic Breeding of the Ministry of Agriculture, Institute of Oil Crops, Chinese Academy of Agricultural Sciences, Wuhan, China; 2 School of Life Sciences, Wuhan University, Wuhan, China; 3 Division of Biological Sciences, University of Missouri-Columbia, Columbia, Missouri, United States of America; 4 National Key Laboratory of Crop Genetic Improvement, School of Life Sciences and Technology, Huazhong Agricultural University, Wuhan, China; East Carolina University, United States of America

## Abstract

*BnAP2*, an *APETALA2* (*AP2*)-like gene, has been isolated from *Brassica napus* cultivar Zhongshuang 9. The cDNA of *BnAP2*, with 1, 299 bp in length, encoded a transcription factor comprising of 432 amino acid residues. Results from complementary experiment indicated that *BnAP2* was completely capable of restoring the phenotype of *Arabidopsis ap2-11* mutant. Together with the sequence and expression data, the complementation data suggested that *BnAP2* encodes the ortholog of *AtAP2*. To address the transcriptional activation of *BnAP2*, we performed transactivation assays in yeast. Fusion protein of BnAP2 with GAL4 DNA binding domain strongly activated transcription in yeast, and the transactivating activity of BnAP2 was localized to the N-terminal 100 amino acids. To further study the function of *BnAP2* involved in the phenotype of *B. napus*, we used a transgenic approach that involved targeted RNA interference (RNAi) repression induced by ihp-RNA. Floral various phenotype defectives and reduced female fertility were observed in *B. napus BnAP2*-RNAi lines. Loss of the function of *BnAP2* gene also resulted in delayed sepal abscission and senescence with the ethylene-independent pathway. In the strong *BnAP2*-RNAi lines, seeds showed defects in shape, structure and development and larger size. Strong *BnAP2*-RNAi and wild-type seeds initially did not display a significant difference in morphology at 10 DAF, but the development of *BnAP2*-RNAi seeds was slower than that of wild type at 20 DAF, and further at 30 DAF, wild-type seeds were essentially at their final size, whereas *BnAP2*-RNAi seeds stopped growing and developing and gradually withered.

## Introduction

Flower is the most important organ of a flowering plant. As a close relative of *Arabidopsis thaliana*, *Brassica napus* has equally a concentric arrangement of four types of flower organs: four sepals in whorl 1, four petals in whorl 2, six stamens in whorl 3, and two fused carpels in whorl 4. Floral organ identity is specified by the transcription factors encoded by 3 classes of floral homeotic genes: the A, B and C functional genes [1]–[3]. Class A genes specify sepals and also interact with class B genes to specify petals. Class C genes specify carpels and also interact with class B genes to specify stamens. Classes A and C genes act antagonistically to restrict each other's activities in perianth and reproductive organs respectively.

*APETALA2* (*AP2*) gene is one of the primary members of class A genes in *Arabidopsis*, characterized by the AP2 DNA binding domain of transcription factors unique to plants, it specifies whorl 1 and 2 organ identity in *A. thaliana* [4], [5]. During floral development, *AP2* is essential for the determination of the identity of sepals and petals. In the weak *ap2* mutants, leaf-like organs replace sepals, and petals exhibit antheroid characteristics, and in the strong *ap2* mutants, carpels are formed in the outer whorl of the flower, petals are absent, and the number of stamens is reduced [6]–[9]. It is especially valuable for oilseed rape (*B. napus*) breeding that *AP2* is also involved in ovule and seed development [5], [10], regulation of seed size [11], [12], and the maintenance of the stem cell niche of the shoot meristem [13]. It has been shown that *AP2* and its closest homologs are the targets of miR172, which down regulates these target genes by a translational inhibition mechanism rather than by RNA cleavage [14].

*AP2* homologs that share similarities in gene structure and function with *AP2* have been isolated from numerous species. The putative *Petunia hybrida* ortholog, *AP2a*, is capable of complementing *ap2* mutants of *Arabidopsis*. In *Petunia*, however, knockout mutations of *AP2a* did not affect floral organ development, suggesting that *AP2* function is redundant in this species [15]. Similarly, two close homologs, *LIP1* (*LIPLESS1*) and *LIP2*, have been identified in *Antirrhinum majus*, both of which need to be inactivated to get an *ap2*-like phenotype [16]. Recently, the closest tomato homolog of *AP2*, *AP2a*, plays a critical role in fruit ripening via regulation of ethylene biosynthesis and signaling [17].

RNA interference (RNAi) is a mechanism of RNA based post-transcriptional gene silencing (PTGS) in eukaryotic cells and has been routinely applied to characterize the gene function and to engineer the novel phenotypes in model plants as well as in cultivated plants. With the recent discovery of gene expression control via small interfering RNA (siRNA) and micro RNA (miRNA) molecules, biologists are exploring genes and development from a new perspective [18]. Understanding of this ubiquitous phenomenon has revealed that RNA interference (RNAi) was a powerful tool to manipulate gene expression and to analyze gene function [19], [20]. Gene constructs encoding direct inverted repeats of the target gene with an intervening functional intron (commonly referred to as intron interrupted hairpin RNA, ihp-RNA) have been shown to induce PTGS with almost 100% efficiency when directed against viruses or endogenous genes [21]–[25], and are more efficient compared to either antisense or co-suppression [26], [27].

For *B. napus*, no *ap2*-like mutants have been identified so far. In this study, we reported the isolation, identification and characterization of *B. napus AP2* (*BnAP2*) gene. To determine whether *BnAP2* gene represented the *A. thaliana AP2* (*AtAP2*) ortholog, we compared *BnAP2* with the complete set of AP2-domain genes in Arabidopsis using the MEGA5.0 software package, analyzed the expression patterns of *BnAP2* in different organs of *B. napus* by RT-PCR. We had further demonstrated the functional conservation of the *BnAP2* by complementing the *ap2-11* mutant of *Arabidopsis* [12]. To study the transcriptional activation of *BnAP2*, we had performed transactivation assays in yeast. Using fusions to the GAL4 DNA binding domain, we showed that the N-terminal of BnAP2 was required and sufficient for transcriptional activation in yeast. Finally, we studied the function of *BnAP2* using the transgenic approach that involved targeted RNA interference repression induced by ihp-RNA in *B. napus*, floral various phenotype defectives and reduced female fertility were observed in *BnAP2*-RNAi lines, loss of the function of *BnAP2* gene also resulted in delayed abscission and senescence of sepals, in the strong *BnAP2*-RNAi lines, seeds showed defects in shape, structure and development and larger size. All the results strongly support the conclusions that *BnAP2* gene is the ortholog of *AtAP2*, and plays a critical role in flower identity and seed development of *B. napus*.

## Results

### Isolation and sequence analysis of *BnAP2*

Total RNA was extracted from the flower buds of *B. napus* cultivar Zhongshuang 9 and SMART cDNA was synthesized using the purified mRNA as a template. According to the known homologous sequence of *A. thaliana*, the specific primers P1 and P2 (Table S1) were designed and the coding sequence (CDS, 1, 299 bp) of *BnAP2* cDNA was obtained from SMART cDNA. The *BnAP2* sequence was submitted to GenBank under the accession number HQ637468.1.

**Figure 1:**
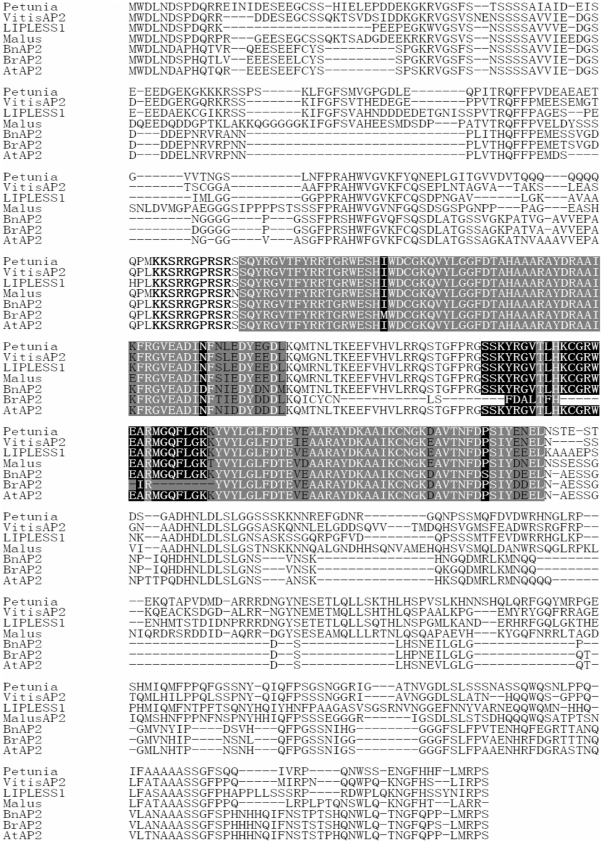
Analysis of the deduced amino acid sequences of *BnAP2*. Comparison of the deduced amino acid sequences of *Brassica napus AP2*-like transcriptional factor gene (*BnAP2*) with other *AP2*-like genes through CLUSTAL X (1.8) multiple sequence alignment software. Comparison of the overall amino acid sequences, the AP2 domains (shaded), linkers, and putative nuclear localization signals (in boldface) of *BnAP2* (GenBank accession number ADU04499.1), *Arabidopsis thaliana APETALA 2* (*AtAP2*) (AEE86718.1), *B. rapa AP2*-like transcriptional factor (*BrAP2*) (AAX47049.1), *Vitis vinifera* transcription factor *APETALA2* (*VitisAP2*) (ACO52508.1), *Malus* x *domestica* transcription factor AHAP2 (*Malus* AP2) (AAL57045.2), *Petunia* x *hybrida* PHAP2A protein (*Petunia* AP2) (AF132001), and *Antirrhinum majus LIPLESS1* (AAO52747.1). Amino acids in the AP2 domains, which are conserved in all mentioned sequences, are in white on a shaded background. The different amino acids in the AP2 domains of BnAP2 and BrAP2 are white on black. Gaps are indicated by dashes.

The *BnAP2* cDNA encoded a protein of 432 amino acid residues (Fig. 1). Similarity searched using tBlastn revealed that the *AtAP2* protein is the most homologous entry in the GenBank database. *BnAP2* showed 86% overall amino acid identity with *AtAP2* and 80% identity with *Brassica rapa AP2* (*BrAP2*). Only full-length sequences with at least 53% amino acid identity were used for further sequence comparison by CLUSTAL X. All possessed high levels of sequence similarity in the double AP2 domain regions (Fig. 1). In addition, the similarity of selected sequences were not limited to the AP2 domains but extended through the reading frame of these genes. A serine-rich putative transcription activation domain (amino acids 16 to 46) [5] and the linker that connected AP2 domains, are conserved, and a putative nuclear localization signal (amino acids 123 to 132) [5] is completely conserved at the amino acid level (Fig. 1). Interestingly, although the relationship of *B. napus* and *B. rapa* is closer than the other species selected, the levels of sequence similarity of the AP2 domains and the linker are lower (Fig. 1). Nine amino acid substitutions and sixteen amino acids deletions exist in the AP2 domains between *BnAP2* and *BrAP2* genes compared with other *AP2*-(like) genes, seven amino acids substitutions and 16 deletions of 25 full linker sequences of the predicted occurred (Fig. 1), while the amino acids mentioned above are conserved in the other *AP2*-(like) genes. Since *B. napus* is a allotetraploid species resulting from a cross between *B. rapa* and *B. oleracea* [28], these data suggest that the cloned *BnAP2* is the *B. oleracea* copy of *AP2*.

**Figure 2:**
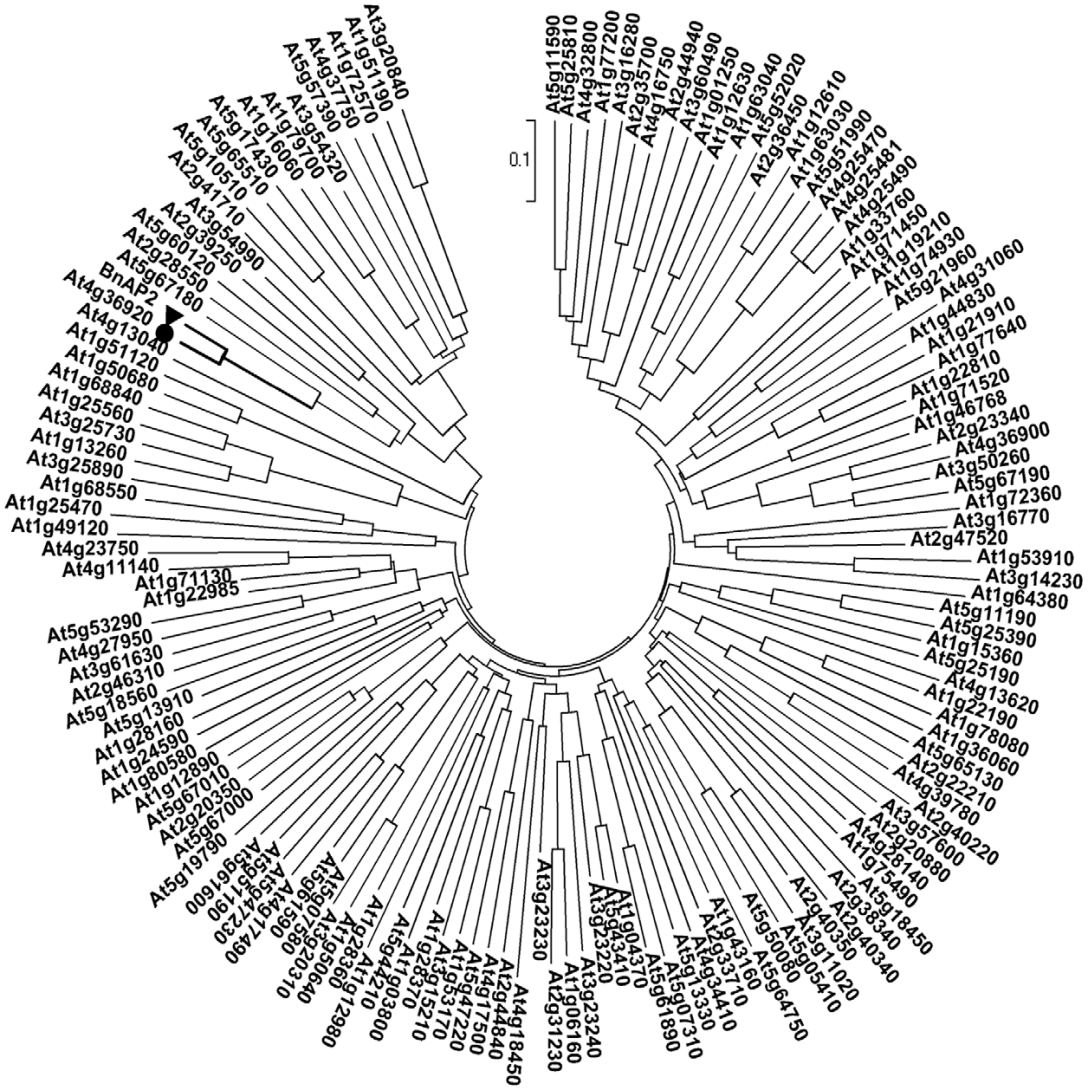
Phylogenetic analysis of *BnAP2* and the complete set of *AP2*-domain in *Arabidopsis*. Phylogenetic relationship was inferred using the Neighbor-Joining method and evolutionary distances were computed using the p-distance method. Assessment of each node confidence was done by means of 1,000 bootstrap replicates. *BnAP2* were denoted with black filled circle and At4g36920, the homolog of *BnAP2* in *A. thaliana* with black filled triangle.

To further evaluate the homology between *BnAP2* and *AP2*-domaingene family in *Arabidopsis*, we compared *BnAP2* with the complete set of *AP2*-domain in *Arabidopsis* using the MEGA5.0 software package. And the result indicated that At4g36920, one member of *AP2/EREBP* gene family in *Arabidopsis*, is one homolog of *BnAP2* in *B. napus* (Fig. 2). In addition, the miR172 target site is also present in *BnAP2* gene through the sequence comparison between *BnAP2* and *AtAP2*. The putative miRNA172 binding site is located within the coding region of *BnAP2* gene but is outside of the conserved *AP2* domains. Nucleotides in *BnAP2* RNA that can base-pair with miRNA172 (with G∶U pairing allowed) are 1178CUGCAGCAUCAUCAGGAUUCU1198.

### The copy number and expression pattern of *BnAP2*

**Figure 3:**
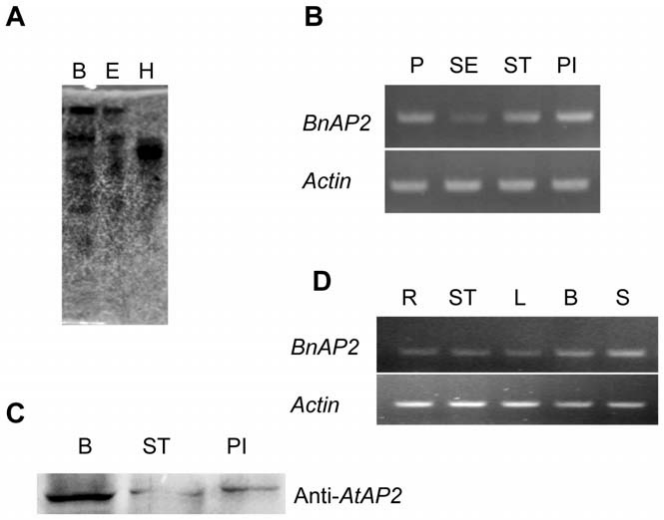
Genomic DNA gel blot analysis and organ-specific expression of *BnAP2* in *Brassica napus*. A, DNA gel blot analysis of the *BnAP2* gene. Genomic DNA (30 ug) was digested with *Bam*HI (B), *Eco*RI (E), and *Hin*dIII (H), and gel separated. The DNA gel blots were hybridized with the full length *BnAP2* cDNA. B, RT-PCR of RNA isolated from *B. napus* (Zhongshuang 6) petal (P), sepal (SE), stamen (ST), and pistil (PI) using gene-specific primers for *BnAP2*. The loading control was *Actin*. C, Western blot analysis of the expression of BnAP2 protein in flower buds (B), stamen (ST), pistil (PI) in *Brassica napus* with the antibody against AtAP2 proein. D, RT-PCR of RNA isolated from *B. napus* (Zhongshuang 6) root (R), stem (ST), leaf (L), bud (B) and silique (S) using gene-specific primers for *BnAP2*. The loading control was *Actin*.

The copy number of the *BnAP2* gene in the *B. napus* genome was estimated by DNA gel blot analysis. *B. napus* genomic DNA was digested with various restriction endonucleases, fractionated, transferred, and hybridized with the CDS of *BnAP2* cDNA as a probe. As shown in Figure 3A, a small number of bands were observed for each DNA digest, indicating that there may be a few copies of *BnAP2* gene in the genome of *B. napus*.

To monitor the expression pattern of the *BnAP2* gene, we isolated total RNAs from different tissues of *B. napus* plant, such as root, stem, leaf, bud, silique (including peel and seed together) and four types of floral organ-sepal, petal, stamen and carpel. By performing reverse transcription-polymerase chain reaction (RT-PCR) analyses with *BnAP2*-specific primer pairs P1 and P3 (Table S1), *BnAP2* gene was clearly expressed at mRNA level in four types of floral organs and thus, functions during the development of all the floral organs (Fig. 3B). Since *AtAP2* is regulated on the protein level by miR172 in whorl 3 and 4, and the miR172 target site is also present in *BnAP2* gene, Western blot analysis of the expression of BnAP2 protein was performed. And the result revealed that BnAP2 was expressed at the protein level in whorl 3 and 4 (Fig. 3C). **A**t present, we could not obtain the antibody against BnAP2 protein, while BnAP2 showed 86% overall amino acid identity with AtAP2, and thus we used AtAP2 antibody, instead of BnAP2 antibody in Western blot. RT-PCR analyses with RNAs from root, stem, leaf, bud and silique indicated that *BnAP2* gene was expressed in all these tissues, and detected weakly in root, stem and leaf, abundantly in bud and silique (Fig. 3D). These results showed that *BnAP2* gene was involved in more global function, in addition to flower identity.

### *BnAP2* complements the *ap2-11* mutant of *Arabidopsis*

**Figure 4:**
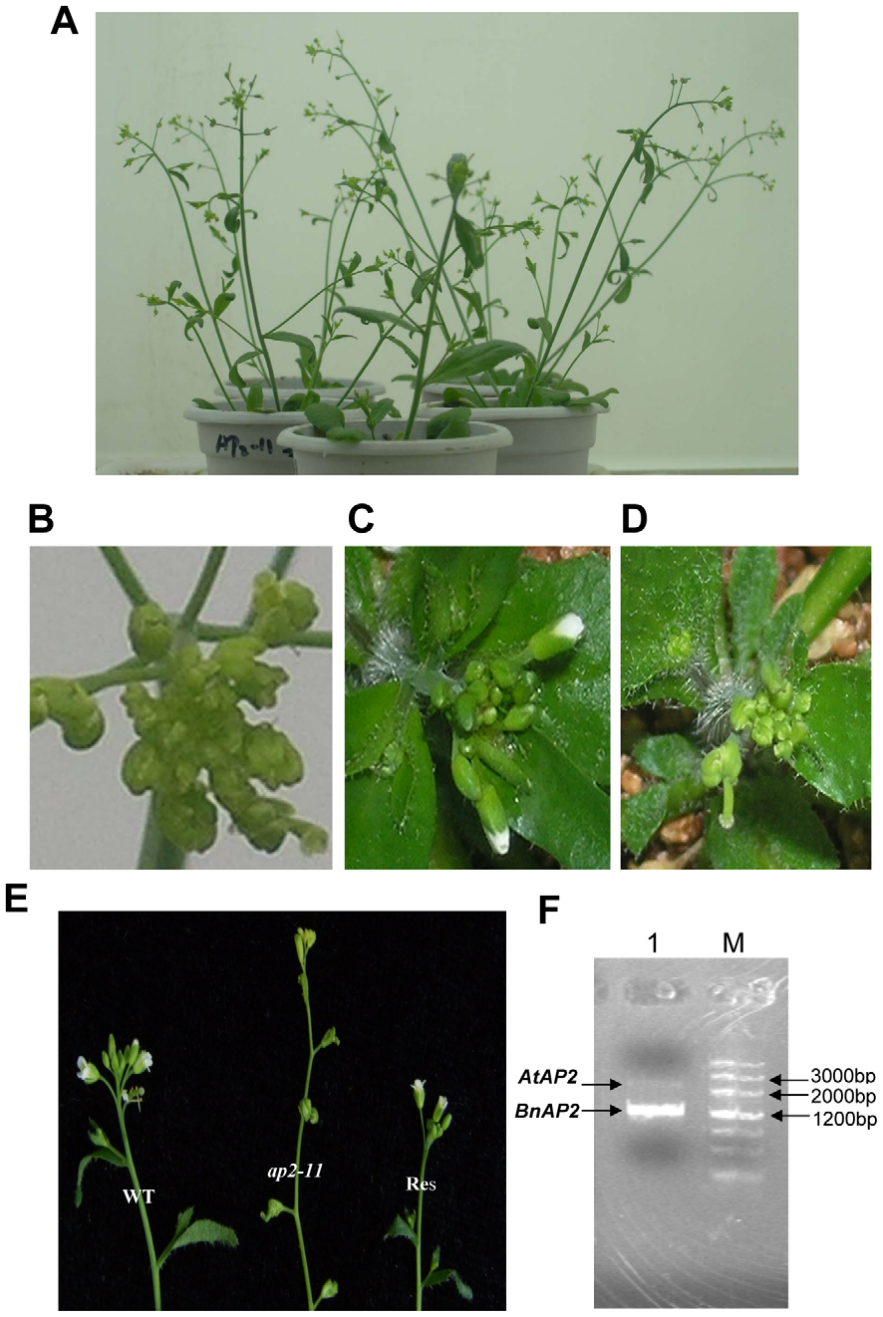
Complementation of the *Arabidopsis ap2-11* mutant by *BnAP2*. *Arabidopsis ap2-11* flower has abnormal phenotype. A, *Arabidopsis ap2-11* mutant plants. B, The inflorescence of *Arabidopsis ap2-11* mutant. C, Flower phenotypes of restoration plants of *Arabidopsis ap2-11* mutant transformed by 35S::*BnAP2*. D, Flower phenotypes of *Arabidopsis ap2-11* mutant. E, The inflorescences of wild type (WT), *ap2-11* mutant (*ap2-11*) and phenotypic restoration (Re) plants. F, PCR analysis of the pBI121-*BnAP2* construct with primers P6 and P7, using *Arabidopsis* transgenic plant genomic DNA as the template. Lane 1: PCR amplification bands for *AtAP2* (2860 bp) and *BnAP2* (1299 bp); Lane M: DNA marker III.

To further identify *BnAP2* as the functional ortholog of *AtAP2*, we decided to express *BnAP2* in the *ap2-11* mutant of *Arabidopsis* to determine whether this might result in complementation. The *ap2-11* mutant resulted from an 11-bp deletion in the *AP2* gene (bases + 724 to + 734 relative to the transcription start site, GenBank accession no. U12546), this region encodes the first AP2 domain of the protein. The strong *ap2-11* mutant had floral defects similar to those of an *ap2* mutant [7], [9]. Sepals were transformed into carpeloid organs, petals failed to develop in the second whorl, and stamen number was reduced. The binary vector pBI121-35S *BnAP2* was used to transform the *ap2-11* mutant (see Methods). All the five independent transformants generated phenotypically wild-type flowers (four sepals, four petals, six stamens) (Fig. 4). Together with the sequence and expression data, the complementation data proved that *BnAP2* encodes the *AtAP2* ortholog in *B. napus*.

### The *BnAP2* protein has transcriptional activation activity in yeast

**Figure 5:**
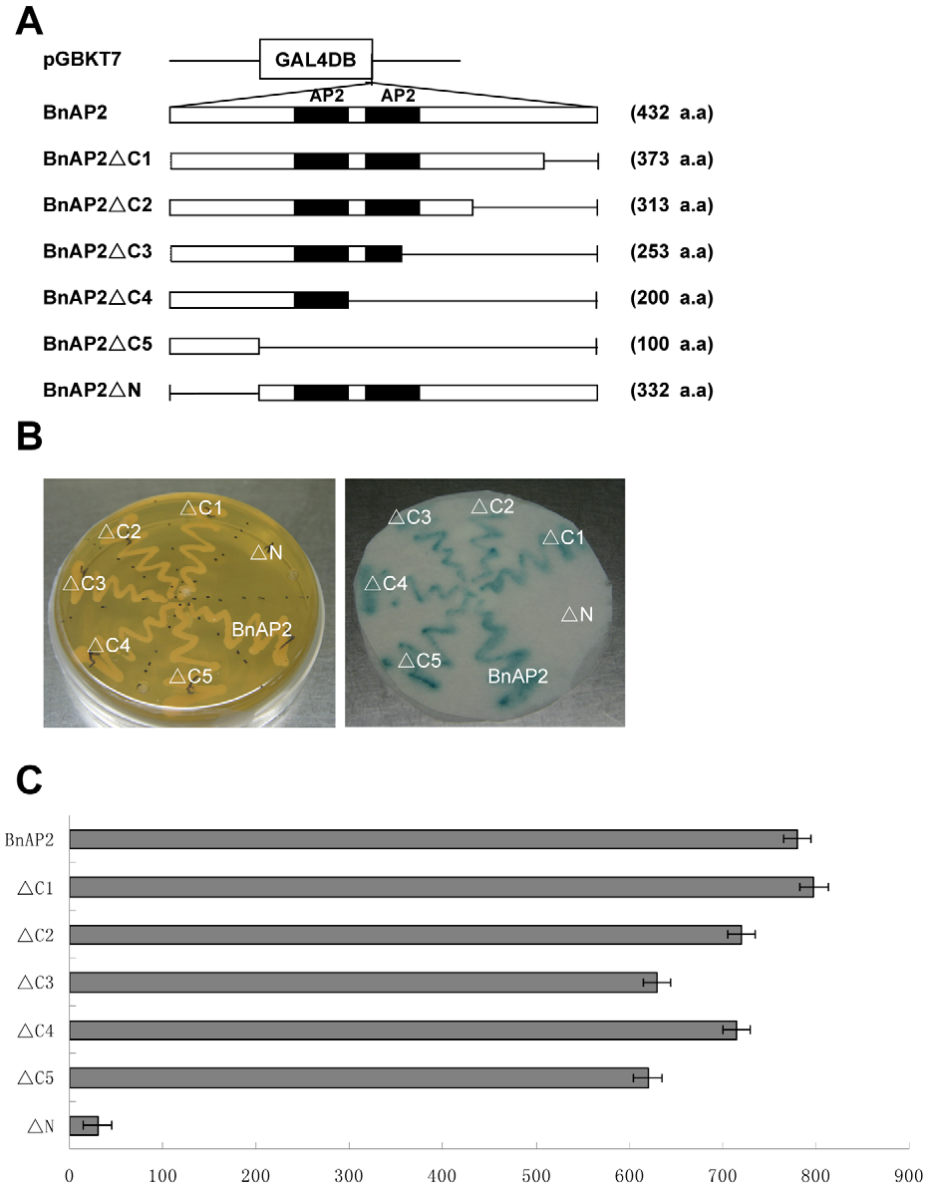
Transcriptional activation activity of *BnAP2* in yeast cells. The transcriptional activity of *BnAP2* was determined using a yeast assay system in which *BnAP2* was expressed in yeast strain AH109 containing a *lacZ* reporter gene. A, *BnAP2* constructs. Six constructs which were fused in frame to the GAL4 DNA binding domain (DB) expression vector were prepared containing various portions of *BnAP2*. B, Filter lift *β*-galactosidase assay was performed. C, The liquid *β*-galactosidase assay was performed using ONPG as a substrate. *β*-Galactosidase activity was then assayed and expressed in Miller units. For each construct, 4 different transformants were assayed. Bars represent standard error.

As shown in Figure 1, by comparison with other *AP2* -(like) genes, *BnAP2* has a serine-rich domain that might act as putative transcription activation domain (amino acids 16 to 46) [5]. To investigate the functional role of this region of *BnAP2*, we fused the CDS for *BnAP2* and its mutants to the GAL4 DNA binding domain expression vector (Fig. 5A) and examined the behavior of each construct as a potential transcriptional activator in yeast (Fig. 5, B and C). In the absence of the GAL4 activation domain, the wild-type BnAP2 protein fused to the GAL4 DNA binding domain activated the transcription of *lacZ* reporter gene. This result indicated that the BnAP2 protein was capable of functioning as a transcriptional activator in yeast. To identify a minimal transcriptional activation domain of BnAP2, various deletion mutants of BnAP2 also were tested in the same manner. The BnAP2ΔC1, BnAP2ΔC2, BnAP2ΔC3, BnAP2ΔC4 and BnAP2ΔC5 mutants, which lacked 59, 119, 179, 232, 332 C-terminal amino acids, respectively, showed almost complete *β*-galactosidase activity (Fig. 5, B and C), while BnAP2ΔN mutant which lacked 100 N-terminal amino acids, demonstrated absence of complete *β*-galactosidase activity (Fig. 5, B and C), indicating that the N-terminal 100 amino acids of BnAP2 play an important role in supporting the ability of BnAP2 as a potential transcriptional activator.

### Generation of the stably inherited *BnAP2*-RNAi transgenic *B. napus* plants

**Figure 6:**
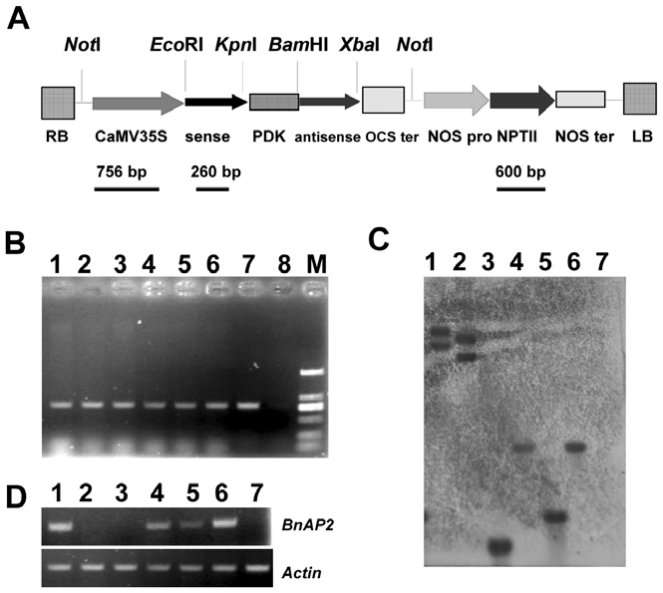
Transformation of *B. napus* cultivar Zhongshuang 6 with intron-spliced hairpin RNA (ihpRNA) as RNAi construct that targets *BnAP2* gene. A, Schematic diagram of T-DNA of the binary vector pBnAP2-RNAi. RB, right border; CaMV 35S, CaMV 35S promoter; Anti-sense, fragment in anti-sense orientation; PDK, PDK intron; sense, fragment in sense orientation; OCS ter, OCS terminator; NOS pro, NOS promoter; NPT II, neomycin phosphotransferase gene; NOS ter, NOS terminator; LB, left border. The sites of a 756-bp probe used for Southern blot, a 600-bp fragment used for PCR template and a 260-bp used for RT-PCR were indicated. B, PCR analysis of the RNAi construct transgenic plants using genomic DNA as the template. Lane 1, positive control (pBnAP2-RNAi); lane 8, untransformed Zhongshuang 6 (CK1); lanes 2–7, the tested transgenic plants *BnAP2*-RNAi-1, *BnAP2*-RNAi-2, *BnAP2*-RNAi-3, *BnAP2*-RNAi-4, *BnAP2*-RNAi-18 and *BnAP2*-RNAi-26, respectively; M, DL 2000 marker. C, Southern-blot analysis of the *BnAP2*-RNAi transgenic plants. Lane M, DNA marker (unit, bp); lane 7, untransformed Zhongshuang 6 (CK1); lanes 1–6, the tested transgenic plants *BnAP2*-RNAi-1, *BnAP2*-RNAi-2, *BnAP2*-RNAi-3, *BnAP2*-RNAi-4, *BnAP2*-RNAi-18 and *BnAP2*-RNAi-26, respectively. Genomic DNA from all transgenic and CK1 plants was digested by *Kpn*I. D, RT-PCR analysis of the *BnAP2* gene in the *BnAP2*-RNAi transgenic plants. lane 1, untransformed Zhongshuang 6 (CK1); lanes 2–7, the tested transgenic plants *BnAP2*-RNAi-26, *BnAP2*-RNAi-18, *BnAP2*-RNAi-4, *BnAP2*-RNAi-3, *BnAP2*-RNAi-2 and *BnAP2*-RNAi-1, respectively.

We constructed an RNAi vector targeting a 5′ terminal 400-bp cDNA fragment of *BnAP2*. The resulting construct, designated *BnAP2*-RNAi, contained the sequence encoding N-terminal transcriptional activation region in an inverted repeat orientation under the transcriptional control of the cauliflower mosaic virus 35S promoter (Fig. 6A). Transformation was performed as the method reported by De Block et al. [29]. Kanamycin-resistant regenerated plantlets that were phenotypically identical to untransformed plants, rooted well in selective medium, were firstly subjected to PCR-based screening with *NPTII* gene specific primers P19 and P20 (Table S1). A total of 6 independent *BnAP2*-RNAi transgenic lines were obtained (Fig. 6B). The transgenic nature for each of them was further confirmed by Southern-blot analysis (Fig. 6C). The results indicated that the transgenic lines *BnAP2*-RNAi-3, *BnAP2*-RNAi-4, *BnAP2*-RNAi-18 and *BnAP2*-RNAi-26 had a single copy of the transgene, and the transgenic lines *BnAP2*-RNAi-1 and *BnAP2*-RNAi-2 had two copies of the transgene. In the second generation, the lines *BnAP2*-RNAi-3, *BnAP2*-RNAi-4, *BnAP2*-RNAi-18 and *BnAP2*-RNAi-26 segregated in a 3∶1 ratio for the transgene as determined by PCR detection. For the lines *BnAP2*-RNAi-1 and *BnAP2*-RNAi-2, the ratio was 15∶1. From the fourth generation, these lines were no longer segregating for the transgene and thus were considered homozygous. *BnAP2* transcripts could not completely be detected in homozygous transgenic lines *BnAP2*-RNAi-1, *BnAP2*-RNAi-18 and *BnAP2*-RNAi-26 by RT-PCR analyses with *BnAP2*-specific primer pairs, while *BnAP2* gene expression was not decreased in homozygous transgenic lines *BnAP2*-RNAi-2, *BnAP2*-RNAi-3 and *BnAP2*-RNAi-4 (Fig. 6D).

### Floral patterning defects in *BnAP2*-RNAi lines

**Figure 7:**
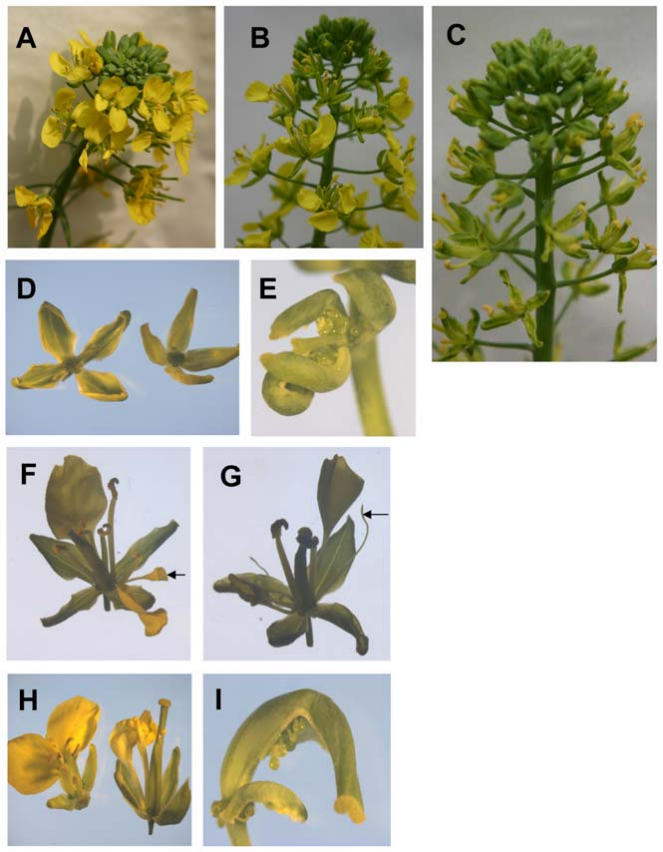
Changes in the number of floral organs and floral identity in the *BnAP2*-RNAi transgenic plants. A, Weakly phenotypic *BnAP2*-RNAi-18 transgenic plant: only involved in reduced numbers of petals in whorl 2 and stamens in whorl 3. B and C, Loss of floral determinacy in severely phenotypic *BnAP2*-RNAi-26 and *BnAP2*-RNAi-1 transgenic plants. D–I, Loss of floral determinacy in severe *BnAP2*-RNAi transgenic plants. D, Sepals were greatly increased in size, both in length and in width, showing distinct leaf-like characteristics, such as a thinner blade in which venation could be easily observed. E, Sepals were fused along the margins, curled at the tip and then transformed into carpeloid organs carrying multiple ovules along the margin. F, Petals formed a aberrant bell-like structure (arrow). G, Stamens converted to filamentous structures (arrow). H, The size of carpel in *BnAP2*-RNAi transgenic plant was somewhat longer than that in wild type plant (right, wild type; left, *BnAP2*-RNAi transgenic plant). I, Misshapen and bent carpel.

Figure 7 shows representative defective phenotypes of flowers in homozygous *BnAP2*-RNAi lines. Out of six analyzed transformants, one *BnAP2*-RNAi line, *BnAP2*-RNAi-18, shows a milder phenotype (Fig. 7A), and two *BnAP2*-RNAi lines, *BnAP2*-RNAi-1 and *BnAP2*-RNAi-26, exhibit severe alterations in flower identity and development (Fig. 7, B and C), and the other three *BnAP2*-RNAi lines, *BnAP2*-RNAi-2, *BnAP2*-RNAi-3 and *BnAP2*-RNAi-4, show no obvious floral patterning defects (data not shown).

As for strong floral defects, the sepals in the first whorl were greatly increased in size, both in length and in width, showing distinct leaf-like characteristics, such as a thinner blade in which venation could be easily observed (Fig. 7D). In addition, the number of sepals reduced, some sepals were fused along the margins, curled at the tip and then transformed into carpeloid organs carrying multiple ovules along the margin, in the late arising or distal flowers along the primary inflorescence (Fig. 7E). The second whorl petals also exhibited dramatic defects. The petals were either absent or reduced in number from four in wild type to between one and three organs. Frequently the petals within a single flower were variable in shape, which formed an aberrant bell-like structure (Fig. 7F). The visible phenotypic defects were in the third whorl of the flower where some stamens were frequently converted to filamentous structures (average 1.59 filaments/flower in *BnAP2*-RNAi-1 and average 1.68 filaments/flower in *BnAP2*-RNAi-26, Table 1) that resembled stamen filaments, but had no anther-like structure at the distal end of the filament (Fig. 7G). In most cases, the structures of stamens were morphologically normal, but the number reduced. The average number of organs that developed in third whorl positions was 2.34 in the line *BnAP2*-RNAi-1 and 2.57 in the line *BnAP2*-RNAi-26 (wild-type average 6 organs), which indicates that there was a frequent failure of organ development in the third whorl of strong *BnAP2*-RNAi lines (Table 1). Most of the fourth whorl carpel were morphologically abnormal, the size was somewhat longer (Fig. 7H), and occasionally misshapen and bent carpels were observed in the late arising or distal flowers (Fig. 7I).

**Table 1:** Average flower organs No. of per flower in BnAP2-RNAi transgenic plants.

	Average No. of Whorl 1		Average No. of Whorl 2		Average No. of Whorl 3	
Genotype	Sepals	Carpeloid Sepals	Petals	Bell-like Petals	Stamens	Filament
*BnAP2*-RNAi-1	2.92	0.85	2.19	1.21	2.34	1.59
*BnAP2*-RNAi-26	2.64	1.07	2.87	1.54	2.57	1.68
*BnAP2*-RNAi-18	4	0	3.12	0.87	6	0
Wild type	4	0	4	0	6	0

In the weak line *BnAP2*-RNAi-18, floral defects only focused on the whorl 2 and 3 floral organs, including that petals were aberrant in number and shape, stamens number was reduced. Table 1 summarized average floral organ number of per flower in above *BnAP2*-RNAi transgenic plants.

### Reduced fertility in *BnAP2*-RNAi transgenic plants

**Figure 8:**
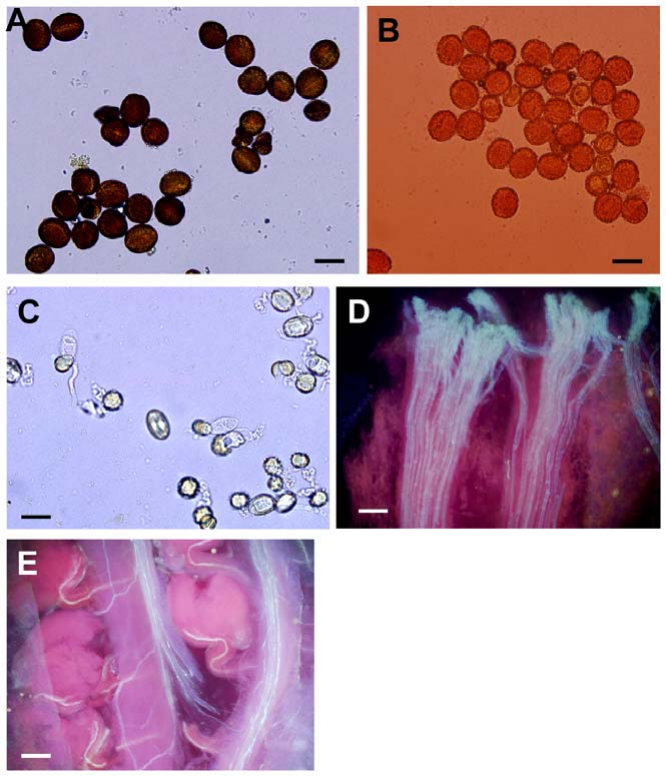
Determination of strong *BnAP2*-RNAi pollen viability, fertility and quality. A, Pollen grains stained with I2-KI. B, Viability of pollen by aceto-carmine staining. C, Pollen germination in vitro. D, Pollen germination on stigma stained with aniline blue. E, Germinating pollen tubes enter into ovules. Scale bars = 50 µm.

*BnAP2* RNAi also induced other defects in reproductive development. Although flower number on the primary inflorescences of mutant plants was similar to that of wild type, fertility was negatively affected by *BnAP2*-RNAi lines. For example, Table 2 shows that strong *BnAP2*-RNAi mutants produced fewer elongated siliques on the primary inflorescence compared with wild type. Pistils failed to elongate into siliques when ovules within the pistil had not been fertilized to a significant extent [30]. Consistent with this result, average seed number per silique was lower in RNAi lines as compared with that of wild type. The *BnAP2*-RNAi mutants produced fewer seeds than wild type because of defects in fertility caused by reduction in *BnAP2* activity. And the reduced fertility in mutants was most likely a consequence of defects in pollen or pistils. To determine pollen viability, fertility and quality, pollen test using I2-KI staining solution or aceto-carmine showed that there was no significant difference between results from the two staining methods. The average pollen viability rate of *BnAP2*-RNAi line was 82.76% from I2-KI staining (Fig. 8A) and 81.58% from aceto-carmine staining (Fig. 8B). Pollen germination experiment in vitro showed that pollens from strong *BnAP2*-RNAi line had normal viability (Fig. 8C). Pollen germination on stigma was also confirmed in *BnAP2*-RNAi mutants by observing the germinating pollen tube on the stigmas 4 h after anthesis (Fig. 8D), Moreover, germinating pollen tubes could enter into ovules (Fig. 8E). In reciprocal crosses experiment, male fertility was further assessed using *BnAP2*-RNAi mutant as the pollinator to cross to Zhongshuang 6, and the average number of seeds each silique produced by this cross was normal; female fertility was assessed using *BnAP2*-RNAi mutants as female parent to cross with Zhongshuang 6, the average number seeds each silique produced by this cross was similar to *BnAP2*-RNAi mutants (Table 3). These results showed that knockdown of the expression of *BnAP2* gene had no effect on pollen viability, fertility and quality, and aberrant pistils induced reduced seed set rate in *BnAP2*-RNAi mutants.

**Table 2:** Fertility and seed weight analysis of BnAP2-RNAi plants.

Genotype	Flower No.	Elongated Silique No.	Length Per Silique (cm)	Seed No. Per Silique	Seed Weight*(mg)
Wild type	42±2.7	42±2.7	11.8±5.3	20±2.3	186^A^
*BnAP2*-RNAi-18	45±1.8	40±3.2	10.0±4.2	18±3.4	185^A^
*BnAP2*-RNAi-1	50±5.2	8±3.4	4.0±1.6	6±3.8	274^B^
*BnAP2*-RNAi-26	48±7.3	10±5.1	5.6±1.2	5±2.4	279^B^

All values pertain to the primary inflorescence. Plants were grown concurrently under identical conditions. Similar results were obtained in an independent experiment that was performed in a different season of the year. Means±SD are shown.

* Weight of seeds produced on primary inflorescence is given in mg per 50 seeds. Seed weight values that differ at the 0.05 significance level are labeled with A and B letters. Seed weights for wild type and *BnAP2*-RNAi-18 and values for *BnAP2*-RNAi-1 and *BnAP2*-RNAi-26 are not significantly different, respectively.

**Table 3:** Aberrant pistils leads to reduced fertility in strong BnAP2-RNAi plants.

Parent					
Female	Male	Flower No.	Elongated Silique No.	Length Per Silique (cm)	Seed No.Per Silique
Wild type	Wild type	10	10	12±2.3	19±3.1
	*BnAP2*-RNAi-1	10	10	11.6±3.3	20±1.1
	*BnAP2*-RNAi-26	10	10	11.4±4.5	21±4.2
*BnAP2*-RNAi-1	*BnAP2*-RNAi-1	10	3	6±2.6	7±3.5
	Wild type	10	4	5.8±1.3	3±2.6
*BnAP2*-RNAi-26	*BnAP2*-RNAi-26	10	3	6±1.8	4±2.7
	Wild type	10	4	5±3.2	4.5±1.5

Reciprocal crosses between wild-type plants and strong *ap2* mutant plants were performed on secondary inflorescences. Plants were grown together in the same conditions. Three flowers at identical positions (11th to 13th flowers for wild type or male-sterile mutant, 10th to 15th flowers in *ap2* mutants) were manually pollinated. Two to four inflorescences per plant and four to five plants were used for calculations. Similar results were obtained in an independent experiment that was performed in a different season of the year. Means ± SD are shown.

### Delayed sepal abscission and senescence induced in strong *BnAP2*-RNAi lines

**Figure 9:**
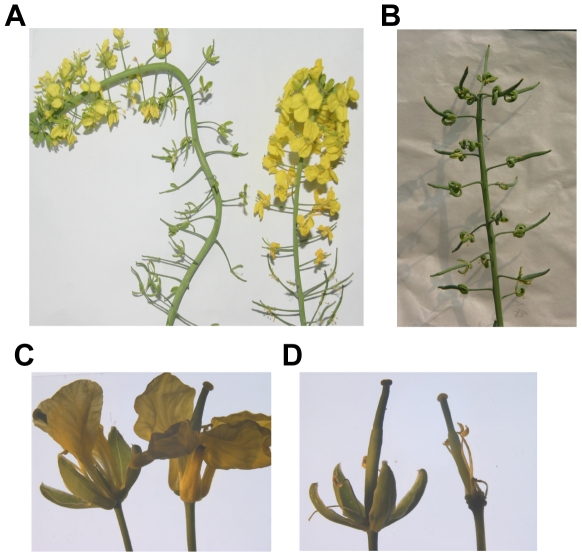
Senescence and abscission were delayed in strong *BnAP2*-RNAi lines. A, Floral organs abscission along the inflorescence in the wild type (WT) (right) and *BnAP2*-RNAi lines (left). B, Sepals in *BnAP2*-RNAi lines retained with silique development. C, On second day following pollination, sepals of the wild type demonstrated yellowing, in contrast, sepals of strong *BnAP2*-RNAi lines keep green. D, On third day following pollination, sepals of the wild type were detached, in contrast, sepals of strong *BnAP2*-RNAi lines remained.

After anthesis occurs in wild-type plants, the sepals, petals, and stamens normally abscise in a short period. As shown in Figure 9A, the floral organs were detached from the siliques in about 3 days after pollination in wild-type *B. napus* plants. In contrast, in strong *BnAP2*-RNAi lines, although the petals and stamens normally abscised following pollination, the sepals remained attached closely at almost all positions of the inflorescence (Fig. 9A), even during later silique development (Fig. 9B).

Senescence usually occurs synchronously with the abscission process in wild-type plants. Therefore, in some mutants with abscission-delayed genetic modifications, such as the *Arabidopsis etr1-1* mutant, the senescence process is also slowed down [31]. We examined the timing of sepal yellowing and the timing of floral organ withering in the wild type and the *BnAP2*-RNAi lines. Just like *etr1-1*, the sepals of the *BnAP2*-RNAi lines showed a blocked progression of senescence compared with that of the wild type that showed yellowing on second day after pollination and withering in 3 days after pollination (Fig. 9, C and D). In contrast, sepals of *BnAP2*-RNAi lines kept green following pollination, even with the whole progress of silique development (Fig. 9B).

### The strong *BnAP2*-RNAi plants are sensitive to ethylene

**Figure 10:**
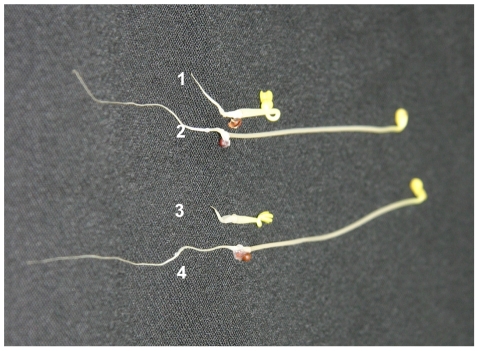
Triple response phenotype of wild-type and strong *BnAP2*-RNAi plants. The triple-response assay was conducted in growing seedlings of wild-type and strong *BnAP2*-RNAi-26 plants on vertical half-strength Murashige and Skoog (MS) agar plates in the absence or presence of 5 mM ACC at 25°C in the dark for 3 d. 1, Seedling of wild-type plant in MS with ACC; 2, Seedling of wild-type plant in MS without ACC; 3, Seedling of *BnAP2*-RNAi-26 plant in MS with ACC; 4, Seedling of *BnAP2*-RNAi-26 plant in MS without ACC.

Abscission processes have been divided into ethylene-dependent and ethylene-independent types [31]. To test the ethylene sensitivity of strong *BnAP2*-RNAi plants, a typical “triple-response” assay [32] was used to determine whether strong *BnAP2*-RNAi plants have normal ethylene perception and response. Seeds germinated vertically in the dark on growth medium supplemented with 5 mM 1-aminocyclopropane-1-carboxylic acid (ACC), which is the natural precursor of ethylene. The strong *BnAP2*-RNAi plants displayed similar triple-response morphological changes compared with the wild-type plants, including the inhibition of hypocotyl growth and root elongation, radical swelling of the hypocotyls, and exaggeration of the curvature of the apical hook (Fig. 10). Therefore, the strong *BnAP2*-RNAi seedlings did not show any deficiency in the perception and response to ethylene.

### *BnAP2* is involved in seed development

**Figure 11:**
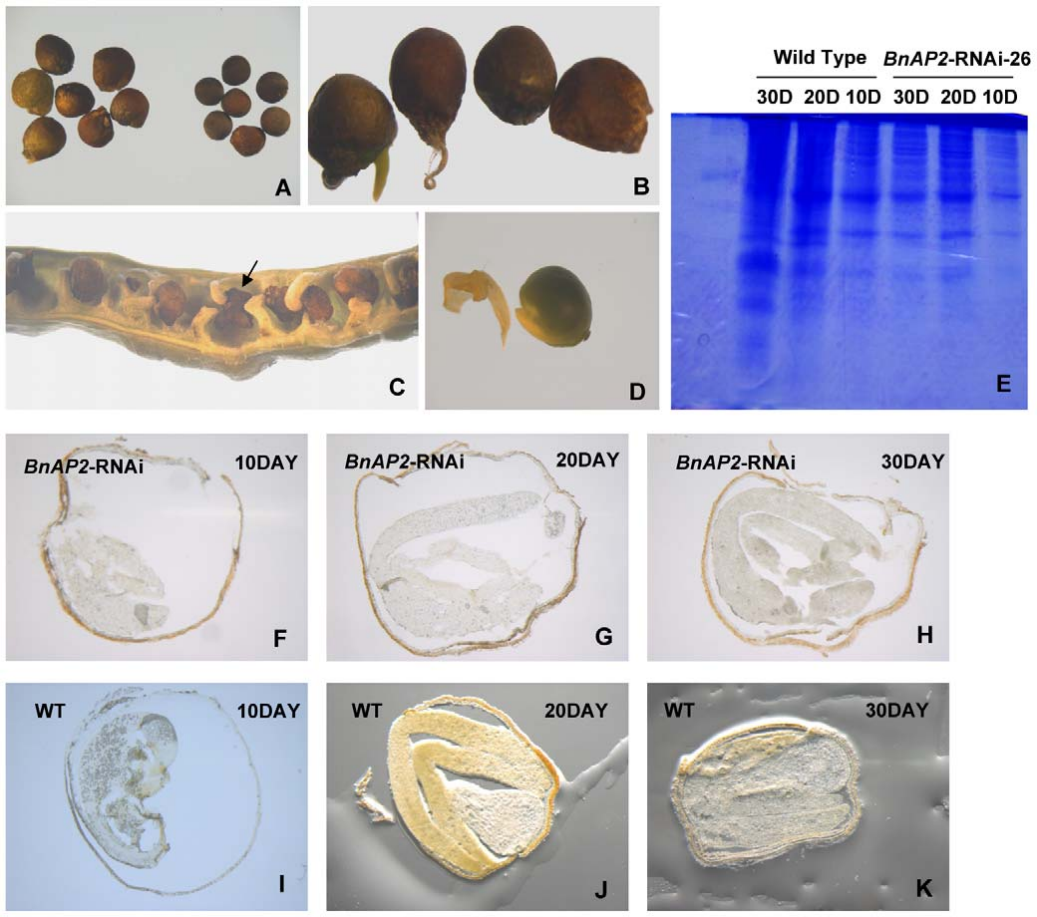
Strong *BnAP2*-RNAi lines cause changes in seed development. A, Seeds of *BnAP2*-RNAi lines are less round in shape and larger in size than that of wild type (left, *BnAP2*-RNAi; right, wild type). B, The surface of the full *BnAP2*-RNAi seed is bumpy and radicels drill out from mature seeds. C, Abortive seeds in strong *BnAP2*-RNAi lines (arrow). D, Embryo of abortive seeds (left, *BnAP2*-RNAi; right, wild type). E, *BnAP2*-RNAi-26 plant seeds contain less protein than do wild-type seeds. Protein extracts from an equal number of wild-type and *BnAP2*-RNAi-26 seeds were fractionated on a 12% SDS polyacrylamide gel and stained. Molecular mass markers are shown to the left of the gel. Optical sections through developing *BnAP2*-RNAi (F–H) and wild-type seeds (I–K) at the indicated DAP. Seeds from three to five siliques at each time point were harvested, fixed and cleared and representative optical sections are shown.

Except floral defects, mild *BnAP2*-RNAi lines did not visibly exhibit other defects under standard growth conditions. However, we found that the seeds of self-pollinated strong *BnAP2*-RNAi lines were structurally and developmentally defective by comparing wild-type and mutant seed. As shown in Figure 11, seeds of strong *BnAP2*-RNAi lines had defects in shape, size, structure and development. Compared with the wild type, the fully mature seeds from *BnAP2*-RNAi-1 and *BnAP2*-RNAi-26 lines had a distorted shape, exhibiting strong indentations resulting in less round seed (Fig. 11A). The surface of the fully mature *BnAP2*-RNAi seed was bumpy, and frequently, radicels drilled out from mature seeds (Fig. 11B), causing dry seeds to fail in germination (data not shown). Additionally, mature seeds of *BnAP2*-RNAi-1 line were larger in size than those of wild type (Fig. 11A). However, the abortive seeds in strong *BnAP2*-RNAi lines were especially interesting, which were abnormal in shape and empty (Fig. 11C). The embryo of abortive seeds was also abnormal in morphology (Fig. 11D). We compared the morphological and developmental changes between abortive and wild-type seeds. Figure 11E shows that there were no obvious changes in total seed protein content in abortive seeds at 10 DAF, 20 DAF and 30 DAF (days after fertilization), and protein accumulation stagnated at 20 DAF, whereas total protein content of wild type seeds increased gradually. Figure 11F-K shows longitudinal sections through cleared seeds at differently developmental stage, including at 10 DAF, 20 DAF and 30 DAF, respectively. Strong *BnAP2*-RNAi and wild-type seeds initially did not display a significantly morphological difference at 10 DAF, suggesting female gametophytes of both genotypes were fertilized with similar efficiencies (Fig. 11, F and I). At 20 DAF, development of *BnAP2*-RNAi embryos was slower than wild type (Fig. 11, G and J). At 30 DAF, wild-type embryos were essentially at their final size (Fig. 11K), whereas *BnAP2*-RNAi embryos stopped growing and developing and gradually withered (Fig. 11H).

## Discussion

### *BnAP2* encodes an *AtAP2* ortholog

In this study, we reported the isolation and partial characterization of an *AP2*-like gene from *B. napus*, here named *BnAP2*. Sequence analysis and phylogenetic tree analysis showed that the BnAP2 protein was the closest AP2 homolog described so far and the homology between BnAP2 and AP2 extends throughout the whole protein. *BnAP2* are broadly expressed in all four types of floral organs and other vegetative different tissues. Moreover, the expression in an *ap2-11* mutant background in *Arabidopsis* resulted in functional complementation. Fusion protein of BnAP2 with GAL4 DNA binding domain strongly activated transcription in yeast, and the transactivating activity was localized to the 100 N-terminal amino acids of BnAP2. Together with the sequence and expression data, the complementation data and transcriptional activity in yeast indicate that *BnAP2* encodes an *AtAP2* ortholog in *B. napus*.

### Suppression of *BnAP2* gene expression in homozygous *BnAP2*-RNAi lines

Post-transcriptional gene silencing (PTGS) is a widely used gene suppression approach that selectively silences genes in plants and animals [33]. PTGS works through sequence-specific degradation of mRNA through endonucleolytic cleavage followed by exonuclease digestion [34] and is considered to have evolved in plants for protection against pathogenic RNAs [35]. There are increasing reports of constructs specifically designed to express dsRNA in plants, usually in the form of self-complementary hairpin RNA (hpRNA), eliciting a high degree and frequency of PTGS of invading viruses, reporter transgenes, and endogenous genes [21]–[23], [36], [37].

Given that *B. napus* is thought to be a diploidized tetraploid, with many non-repetitive sequences in genome being present in more than two copies, RNAi is expected to be a powerful tool for genetic modification of such multiple-copy genes. Using intron-spliced hairpin RNA (ihpRNA) as RNAi construct that targeted *BnAP2* gene, we totally obtained 6 transgenic lines. Although PCR-based screen and DNA gel blot confirmed the integration of foreign *BnAP2*-RNAi fragment into the genome of *B. napus*, 3 transgenic lines did not exhibit any morphological changes. RT-PCR analysis showed there was no decrease in the mRNA of *BnAP2* in the buds of the corresponding RNAi lines. We speculated that transgenic silence led to the consequence. At the same time, *BnAP2* gene expression could not be detected by RT-PCR in other 3 transgenic plants, which indicated that there was specific and efficient suppression of target gene. Of which, two *BnAP2*-RNAi lines showed significant defects in flower organs and seed development, and one *BnAP2*-RNAi line only showed floral defects. These results demonstrated for the first time, to our knowledge, that ihpRNA constructs targeted against an endogenous target gene were clearly able to generate phenotypic changes in *B. napus*, making this approach an efficient technique for genetic modification of important agronomic traits in oil crops.

### Floral defects and reduced fertility induced in *BnAP2*-RNAi lines

In *Arabidopsis*, the floral homeotic gene *APETALA2* (*AP2*) controls three critical aspects of flower ontogeny, including the establishment of the floral meristem [38]–[42], the specification of floral organ identity [6]–[8], and the temporal and spatial regulation of floral homeotic gene expression [43], [44]. Clearly, *AP2* plays a critical role in the regulation of *Arabidopsis* flower development. Null mutants of the *AP2* gene in *Arabidopsis* confer a mutant phenotype that fit almost perfectly with the A-function in the ABC-model. The phenotypes described in the strong RNAi lines are in agreement with the notion that BnAP2 exert the A-function in *B. napus*. In this study, floral phenotype defectives in *B. napus BnAP2*-RNAi lines were observed, which was similar to those described in *Arabidopsis*. Weak *BnAP2*-RNAi line, having defects in petals and stamens, showed normal fertility. By contrast, two strong *BnAP2*-RNAi lines not only exhibited the loss of floral identity but also reduced fertility. Subsequent pollen stainability, pollen germination and reciprocal cross experiment indicated that pollens from strong *BnAP2*-RNAi lines had normal quality and viability. These results told us that knockdown of *BnAP2* gene had no effect on pollen fertility and induced aberrant pistils maybe had a negative effect on female fertility. Nevertheless, we could not exclude the possibility that reduced fertility of the RNAi lines resulted from the suppression of other similar *AP2* genes, in addition to the *BnAP2* gene.

### *BnAP2* gene is involved in floral organ abscission and senescence

In plants, abscission and senescence events allow the shedding of leaves, flowers, fruits and seeds, and can facilitate growth, reproduction, and defense against pathogens [45], [46]. As with most developmental events, proper timing and spacing are crucial during organ separation [47]. Floral organ abscission is a developmentally controlled program that occurs after flower pollination. Ethylene is considered to be a fundamental regulator of the abscission rate [31], [48]. Defects in the components of the ethylene perception and signaling pathways will delay abscission to various degrees. In *Arabidopsis* ethylene-insensitive mutants, such as *etr1* and *ein2*, both floral organ abscission and senescence are delayed [31], [46]. However, abscission processes have been divided into ethylene-dependent and ethylene-independent types [31], and therefore further experiments would be carried to determine whether the *BnAP2*-RNAi plants have normal ethylene perception and response.

However, ethylene is not the only regulator of abscission, and abscission processes have been divided into ethylene-dependent and ethylene-independent types [31]. A ligand gene family, including INFLORESCENCE DEFICIENT IN ABSCISSION (IDA) and five IDALIKE (IDL) genes, participates in the control of abscission, and its function is not affected by exogenous ethylene [49], [50]. In addition, some genes that relate leaf and floral organ patterning also influence the capacity for abscission. These genes include two well-known leaf-patterning factors, BOP1/BOP2 [51], a MADS box domain transcription factor, AGL15, and two chromatin regulators, ARP4 and ARP7 [52]–[54]. The triple-response assay of wild type and *BnAP2*-RNAi-26 plants confirmed that the *BnAP2*-RNAi plants had normal ethylene perception and response. And the result suggested that *BnAP2* gene had an effect on sepal abscission with the ethylene-independent pathway. At the same time, we could not exclude the possibility that delayed sepal abscission and senescence of the RNAi lines was caused by the inhibition of other similar *AP2* genes, in addition to the *BnAP2* gene.

### Knockdown of *BnAP2* gene expression disordered seed development

Our analysis of *BnAP2* gene expression at the RNA level revealed that *BnAP2* was expressed in both nonfloral and floral tissues and organs. We therefore proposed that *BnAP2* had a more expanded role in *B. napus* growth and development. In strong *B. napus BnAP2*-RNAi lines, dried, full and mature seeds produced through self-pollinating were larger and more variable in shape than circle-formed wild-type seeds because of knockdown of *BnAP2* gene. In addition, some full and mature seed coat development was affected, leading to the radicles exposed to seed coat.

Seed development in higher plants begins with a double fertilization process that occurs within the ovule and ends with a dormant seed primed to become the next plant generation [55], [56]. Many transcription factors (TFs) are responsible for controlling seed development. Le et al. [57] carried global analysis of gene activity during *Arabidopsis* seed development and identified 289 seed-specific genes, including 48 that encode TFs. In *Arabidopsis*, *AP2* was involved in controlling ovule, seed coat development, seed size and seed development [10]–[12], [41], [58]. Then, these results suggested that *BnAP2* gene played an important role in seed development.

Study of abortive seed development in *B. napus BnAP2*-RNAi lines further confirmed above proposal. The seed consists of three major compartments, the embryo, endosperm and seed coat, that originate from different cells of the ovule and possess different complements of maternal and paternal genomes [59]. Seed development proceeds through two distinct phases during which growth of the three compartments is coordinated. During the early morphogenesis phase, the embryo undergoes a series of differentiation events in which the plant body plan is established with the formation of embryonic tissue and organ systems. It is also during this phase that the endosperm mother cell initially undergoes nuclear division without cytokinesis to form a syncytium [60]. Syncytial nuclei are sequestered into individual endosperm cells through the process of cellularization, and the endosperm continues to grow through periclinal cell divisions at the periphery of the endosperm. Later in embryogenesis during the maturation phase, the embryo and endosperm accumulate reserves such as storage lipids and proteins, and the embryo acquires the ability to withstand desiccation [61], [62]. In plants such as *B. napus*, the endosperm is largely consumed by the developing embryo such that only a few endosperm cell layers remain in the mature seed. In *B. napus BnAP2*-RNAi lines, total protein content of abortive seeds showed no conspicuous alterations at 10 DAF, 20 DAF and 30 DAF. This result suggested that there was an obstacle in accumulating reserves such as proteins in embryogenesis during the maturation phase. Frozen section analysis revealed that during the early morphogenesis phase of abortive seeds, the embryo successfully undergoes a series of differentiation events in which the plant body plan was established with the formation of embryonic tissue and organ systems. But embryos established did not successfully develop into normal and full seeds, stopping growing at 20 DAF. Together, loss of *BnAP2* function influenced seed development in embryogenesis during the maturation phase.

In conclusion, as a key transcriptional factor, *BnAP2* gene is involved in the regulation of many target genes and plays essential roles in floral identity, sepal abscission and senescence, and seed development in *B. napus*. Since the RNAi lines, in addition to the *BnAP2* gene, also affected the expression of other similar *AP2* genes, we could not exclude the possibility that reduced fertility and delayed sepal abscission and senescence was led by the suppression of other paralogues. And further experiment would be conducted in order to screen paralogues affected in the RNAi lines through transcriptome profiling. Although knockdown of *BnAP2* gene caused mature and full seeds to be larger than wild seeds, total seed yield could not be raised in *BnAP2*-RNAi lines because of reduced female fertility and abortive seed. However, it is unclear how *BnAP2* acts to affect seed development. Additional experiments are needed to understand how *BnAP2* affects seed development and seed size at a mechanistic level. To unravel the genes that might be directly or indirectly regulated by *BnAP2* in *B. napus*, further research would be focused on comparing gene expression of seed development between wild type and transgenic knockdown plants through transcriptome profiling.

## Materials and Methods

### Isolation and sequencing of the *BnAp2* gene

Total RNA was extracted from the flower buds of *B. napus* cultivar Zhongshuang 9 using the RNeasy Plant Mini Kit (Qiagen, USA) and first-strand cDNA was synthesized following the procedure described by SMART™ PCR cDNA Synthesis Kit (Clontech, TaKaRa) and was used as a template. Two specific primers P1 and P2 (Table S1) were designed based on the known *AP2* gene sequence of *A. thaliana*. PCR reaction was performed as follows, firstly denatured at 94°C for 2 min, then 94°C 30 s, 64°C 30 s, 72°C 90 s for 14 cycles and the annealing temperature decreased 1°C every cycle, then 94°C 30 s, 50°C 30 s, 72°C 90 s for 22 cycles, and then 72°C 5 min. The specific fragment obtained was cloned into the pMD18-T vector (TaKaRa, Japan) and sequenced at Beijing Huada Company (China).

### RT-PCR

Total RNA was isolated from root, shoot, leaf, flower bud, silique (including peel and seed) and four types of floral organs using the RNeasy Plant Mini Kit (Qiagen, USA), respectively. And then, RNA was treated with DNase, extracted with phenol/chloroform, and precipitated, and 0.5 µg of total RNA was reverse transcribed from an oligo-dT primer by using the SuperScript® III First-Strand Synthesis System (Invitrogen). The first-strand cDNA was used as a template for RT-PCR amplification with corresponding primers (Table S1).

### PCR-based screening and southern-blot analysis

Genomic DNA was extracted from fresh leaf tissues of in vitro-grown Kan-positive plants and untransformed control plants by the cetyltrimethylammonium bromide (CTAB) method [63]. For transgenic detection, PCR-based screening was used to maintain the presence of the transgene. Specific primers for the *NptII* gene: P19 and P20 were used (Table S1). These primers were expected to give products of 600 bp. PCR analysis was done according to the method reported by Yu et al. [64]. For Southern blot analysis which confirmed the integration of the target gene, the probe was specific to the 35S promoter. The primers used for the 35S promoter probe were P21 and P22 (Supplemental Table S1), which amplified a 756-bp product. For Southern blot analysis which detected copy number of *BnAP2* gene, the probe was specific to the *BnAP2* gene. The primers used for the *BnAP2* gene probe were P1 and P2 (Table S1), which amplified full *BnAP2* gene product. Total thirty micrograms of genomic DNA were digested with appropriate restriction enzyme. The restriction fragments were size-fractionated by 0.8% (w/v) agarose gel electrophoresis and transferred to a Hybond-N^+^ nylon membrane (Amersham Pharmacia Biotech, UK). The labeling of probe, prehybridization, hybridization and detection were performed following the procedures described by DIG High Prime DNA Labeling and Detection Starter Kit II (Roche Diagnostics GmbH, Germany).

### Vector construction

*BnAP2* gene over-expressing vector used to transform *Arabidopsis* was constructed by PCR using the synthetic oligonucleotide primers P6 and P7 (Supplemental Table S1). The amplified full-length *BnAP2* cDNA fragment was digested with *Bgl*II, then cloned into pBI121 [65], which had been cut with *Bam*HI. The resulting *BnAP2*-expressing vector was designated as pBI121-*BnAP2*.

Vector construction for RNAi knockout, RNAi vector pHANNIBAL [23] was used to generate RNAi constructs. The sense and antisense cDNA sequences of the *BnAP2* gene were amplified from CDS of *BnAP2* cDNA through PCR and placed under the control of a constitutively expressing 35S promoter in pHANNIBAL. The sense fragment was amplified using gene specific primers having restriction sites *Eco*RI (P15) and *Kpn*I (P16) and inserted as an *Eco*RI-*Kpn*I fragment into pHANNIBAL which was cut with *Eco*RI and *Kpn*I (Supplemental Table S1). The antisense fragment was amplified using gene-specific primers having restriction sites *Xba*I (P17) and *Bgl*lII (P18) and was inserted as an inverted fragment as *Xba*I-*Bgl*lII pHANNIBAL which was cut with *Xba*I and *Bam*HI (Supplemental Table S1). The pHANNIBAL vector was then subcloned at *Not*I sites into a binary vector pART27 [66].

For construction of various *BnAP2* deletion mutants and full *BnAP2* for transactivation activity assays, the deletion mutant genes were generated by PCR using restriction sites within the synthetic oligonucleotide primers. The primers were shown in Table S1. The forward primers carried *Eco*RI and the reverse primers had *Bgl*lII. The restriction sites used in the primers to facilitate the cloning of the PCR product into the yeast GAL4 DNA binding domain vector pGBKT7 were underlined. pGBKT7 was cut with *Eco*RI and *Bam*HI. The plasmids pBnAP2ΔC1, pBnAP2ΔC2, pBnAP2ΔC3, pBnAP2ΔC4 and pBnAP2ΔC5, encoded BnAP2 proteins with deletion of 59, 119, 179, 232 and 332 C-terminal amino acids, respectively. The plasmid pBnAP2ΔN1 encoded BnAP2 proteins with deletion of 100 N-terminal amino acids. The plasmid pBnAP2 which encoded full BnAP2 protein was generated by PCR with P6 and P14.

### Plant transformation (*Arabidopsis* complement experiment, *B. napus* transformation)

For *Arabidopsis* transformation, the *Arabidopsis ap2-11* mutant (kindly provided by John J. Harada) was transformed according to the vacuum infiltration method [67] using *Agrobacterium* strain EHA105. T3- or T4-generation homozygous plants were used for phenotype analysis.

*Agrobacterium*-mediated transformation of *B. napus* cultivar Zhongshuang 6 was performed according to the protocol described previously [29]. Kanamycin-resistant plantlets that rooted well in selective medium were transferred to pots and grown in a glasshouse. For plants grown in a plant growth room, the growth conditions were 20±2°C under a 16/8 h photoperiod at a light intensity of 44 umol m^−2^ s^−1^ and 60–90% relative humidity. For plants sown in a glasshouse, the conditions were 14–22°C and natural light.

### Ethylene response

Ethylene treatment was performed as described previously [68]. For the triple response, 5 mM of the ethylene precursor ACC was added to Murashige and Skoog (MS) medium for germination of wild-type or transgenic seeds in the dark for 3 d.

### Transactivation activity assays

For the yeast strains and transformation, the yeast strain *Saccharomyces cerevisiae* strain AH109 (MATa, HIS3, lacZ, ADE2, MEL1) was transformed with plasmids containing CDS of *BnAP2* cDNA or *BnAP2* fragments which were fused in frame to the yeast GAL4 DNA binding domain vector pGBKT7, using a modified lithium acetate procedure [69]. Transformants were selected on plates containing synthetic medium lacking tryptophan. The colony lift filter assay and liquid culture assay using o-nitrophenyl-*β*-D-galactopyranoside as a substrate were performed subsequently as described by the manufacturer (Clontech, Palo Alto, CA) to determine the ability of each translation product to activate transcription.

For the filter lift *β*-galactosidase assay, transformants were streaked onto a synthetic complete supplement (SC)-Trp-His-ADE medium, grown at 30°C for 3 d, and lifted from the medium using filter papers (Whatman No. 5). The filters were immersed into liquid nitrogen for 30 s, thawed at room temperature for 5 min, and placed on filter papers soaked with an X-gal (0.5 mg/ml) solution. The reactions were stopped after 2 to 4 hours at 30°C. For each construct, 3 independent transformants were assayed.

For the liquid *β*-galactosidase assay, colonies of yeast transformants were grown in an SC-Trp-His-ADE medium to an A_600_ of approximately 0.5. The cultures were diluted 4 times with fresh media and grown further for 3 h. After the A_600_ was measured, 1.5-ml aliquots of the cultures briefly underwent centrifugation in a microfuge. The pellets were resuspended in 0.5 ml of Z-buffer (60 mM Na_2_HPO_4_, 40 mM NaH_2_PO_4_, 10 mM KCL, and 1 mM MgSO_4_•7H_2_O) without *β*-mercaptoethanol, microfuged briefly again, and resuspended in 0.3 ml of Z-buffer. One third of the suspension was transferred to a fresh tube, placed in liquid nitrogen until frozen, and thawed in a 37°C water bath. The freeze-thaw cycle was repeated 2 more times. Afterward, 0.7 ml of Z-buffer with *β*-mercaptoethanol and 0.16 ml of o-nitrophenyl-*β*-D-galactopyranoside (ONPG) (4 mg/ml in Z-buffer) were added to start the reaction. Incubation was continued at 30°C until the color changed to yellow. Reactions were stopped by the addition of 0.4 ml of 1 M Na_2_CO_3_. The mixtures underwent microcentrifugation for 10 min to remove cell debris, and the A_420_ was measured. Four independent transformants were assayed for each construct, and the *β*-galactosidase activity was expressed in Miller units.

### Determination of pollen viability, fertility and quality

Pollen viability test was performed with the use of two staining procedures, one reported by Shinjyo [70] and the other by Wei et al. [71], and in vitro pollen germination. Newly opened flowers were sampled at 9–10 a.m. Anthers were squashed and pollen grains were stained with 1% w/v iodine/potassium iodide solution (I2-KI) to observe starch accumulation or with 1% aceto-carmine, and were observed under microscopy. Five individual plants of each material and five flowers of each plant were used and the calculation was based on 200 pollen grains of each flower. Pollen viability rate was calculated as the number of well-stained pollen grains/total pollen grains ×100%. For pollen germination in vitro, pollen grains was isolated from flowers of strong *BnAP2*-RNAi lines and immediately germinated in vitro using Hodgkins and Lyons media containing 9% sucrose and 13% polyethylene glycol (MW 4000) [72]. The pollen was incubated in light and high humidity for 6 h at 23°C. Germinating pollen (those with pollen tubes greater than twice the length of the pollen grain) were counted and photographed. Pollen germination on the stigmas was observed using aniline blue staining as previously described [73]. Briefly, 3 h after pollination, pistils were fixed with ethanol/acetic acid (3∶1 v/v) for 30 min, washed with 1 N KOH at 55°C for 30 min, and stained with 1% w/v aniline blue for 40 min at 37°C. Pollen fertility of *BnAP2* RNAi mutants was also assessed according to reciprocal crosses between Zhongsuang 6 wild-type plants and *BnAP2*-RNAi mutants. Plants were grown together in the same conditions. Flowers at identical positions were manually pollinated. Two to four inflorescences per plant and four to five plants were used for calculations. Similar results were obtained in an independent experiment.

### Protein analysis

Ten seeds from Zhongshuang 6 wild-type plants or *BnAP2*-RNAi lines, at 10 DAY, 20 DAY, 30 DAY, respectively, were homogenized with 100 ul of extraction buffer [74] by using a microglass pestle and mortar, respectively. After centrifugation, 10 ul of each extract was used for SDS-PAGE [75]. Protein content in 5 µl of each extract was determined by using the Bio-Rad RC-DC Protein Assay Kit with BSA as the standard.

### Western blot analysis

Protein extracts of flower buds, floral organs in whirl 3 and 4, respectively, were prepared according to the method described above. The concentration of protein in the supernatant was quantified using a protein assay kit (Bio-Rad). Proteins were separated by 10% sodium dodecyl sulfatepolyacrylamide gel electrophoresis (SDS-PAGE) and electrotransferred to nitrocellulose membranes. The membranes were probed with a goat polyclonal AtAP2 antibody (Santa Cruz), followed by incubation with horseradish peroxidase conjugated donkey anti-goat IgG (Santa Cruz). Immunoreactivity was visualized by chemiluminescent detection (Pierce). Immunoblots were visualized with the ECL detection system (Pierce).

### Phylogenetic Analysis

To detect BnAP2 homolog in *A. thaliana*, all the protein sequences of AP2-domain genes in *A. thaliana* were downloaded from RARTF database (http://rarge.psc.riken.jp/rartf/) [76]. Selected 145 Arabidopsis AP2-domain genes and target BnAP2 were used to constructed phylogenetic trees with the MEGA5.0 software package [77]–[80]. Phylogenetic relationship was inferred using the Neighbor-Joining method and evolutionary distances were computed using the p-distance method.

### Frozen section

Seeds at different developmental phase were fixed with FAA (10% formalin/5% acetic acid/45% ethanol/0.01% Triton X-100) for 45 min, and rehydrated through an ethanol series. Seeds were then embedded in OCT compound and frozen, sectioned at 10-µm thickness with frozen section machine Leica CM1850 (Germany). Observations were made with an Olympus microscope and photographed.

## Supporting Information

Table S1 List of primers used.
(PDF)
