# Correction: Functional Identification and Characterization of the *Brassica Napu*s Transcription Factor Gene *BnAP2*, the Ortholog of *Arabidopsis Thaliana APETALA2*

**DOI:** 10.1371/annotation/8d7e5659-d669-4794-9fb8-40a7e8267a48

**Published:** 2012-08-03

**Authors:** Xiaohong Yan, Lei Zhang, Bo Chen, Zhiyong Xiong, Chunli Chen, Lijun Wang, Jingyin Yu, Changming Lu, Wenhui Wei

The following information was missing from the funding section: National High Technology and Development Program of China (grant no. 2008AA10Z152).

It should also be noted that the correct listing of the corresponding authors should be:

whwei@oilcrops.cn (WW); cmlu@oilcrops.cn (CL) 

