# Supplementary material for: Functional Identification and Characterization of the Brassica Napus Transcription Factor Gene BnAP2, the Ortholog of Arabidopsis Thaliana APETALA2
Source: PLoS One. 2012 Mar 27;7(3):e33890. doi: 10.1371/journal.pone.0033890 (PMC3314020; doi:10.1371/journal.pone.0033890)
Supplement: Table S1 — List of primers used. (PDF) [file pone.0033890.s001.pdf]

Supplemental Table S1. List of primers used.

Primer	Sequence	Purpose of primers
Full-AP2 For (P1)	5’ ATGTGGGATCTAAACGACGC 3’	Amplification of *BnAP2* gene
Full-AP2 Rev (P2)	5’ TTAAGAAGGTCTCATGAGAG 3’	and for probe preparation
AP2-260 Rev (P3)	5’ CGAGGAAAGCTCGACCCGGG 3’	RT-PCR of *BnAP2* gene as reverse primer, P1 as forward primer
Actin For (P4)	5’ TGTATGTTGCTATCCAGGCT 3’	Amplification of *actin* gene as
Actin Rev (P5)	5’ GTGAGAATCTTCATCAGGGA 3’	internal control gene
AP2 For (P6)	5’ACCAGATCTATGTGGGATCTAAACGACGC3’	Construction of restorer
AP2 Rev (P7)	5’GGCAGATCTAGAAGGTCTCATGAGAG3’	vector
△C1 Rev (P8)	5’CCAGAATTCGAGAATCCTCCTCCGCCATG3’	Construction of various
△C2 Rev (P9)	5’GTCGAATTCGTTTCCCAAACTCAAATCGA3’	C-terminal *BnAP2*
△C3 Rev (P10)	5’GTCGAATTCATAAACATACTTTTTGCCTA3’	deletion mutants, P6 as
△C4 Rev (P11)	5’CCGGAATTCCTTCATGTCATTATCATAAT3’	the forward primer
△C5 Rev (P12)	5’GGCGAATTCTAGGTCCGACTGAGAGAACT3’	
AP2△N For (P13)	5’TAAGGATCCAGTTCTCTCAGTCGGACCTA3’	Construction of N-ter
AP2△N Rev (P14)	5’GGCGAATTCTTAAGAAGGTCTCATGAGAG3’	*BnAP2* deletion mutant
AP2i For (P15)	5’ACAGAATTCATGTGGGATCTAAACGACGC3’	Construction of
AP2i Rev (P16)	5’TAAGGTACCAACTCCTTGACCTTGGTCCA3’	*BnAP2* RNAi vector
R-AP2i For (P17)	5’GGCTCTAGAATGTGGGATCTAAACGACGC3’	
R-AP2i Rev (P18)	5’CACAGATCTAACTCCTTGACCTTGGTCCA3’	
NPT II For (P19)	5’GATGGATTGCACGCAGGTTC3’	Amplification of
NPT II Rev (P20)	5’CTTGGACGCACGTTAGGTAG3’	NPT II gene
35S For (P21)	5’AGAATGCTAACCCACAGATG3’	Amplification of
35S Rev (P22)	5’GGATAGTGGGATTGTGCGTC3’	35S gene

The restriction enzyme sites added at the 5’ end of primer sequences are underlined
